# Patterns of alcohol use and response to digital brief interventions in college students: a secondary analysis of a cluster randomized trial

**DOI:** 10.3389/fpsyt.2025.1732518

**Published:** 2026-01-12

**Authors:** Abhishek Ghosh, Blessy B George, Narayanan C Krishnan, Renjith R Pillai, Soundappan Kathirvel, Mamta Sharma, Anil Kumar, Debasish Basu, Michael Patrick Schaub

**Affiliations:** 1 Department of Psychiatry, Post Graduate Institute of Medical Education and Research (PGIMER), Chandigarh, India; 2 Department of Data Science, Indian Institute of Technology Palakkad, Kanjikode, India; 3 Department of Community Medicine and Public Health, Postgraduate Institute of Medical Education and Research, Chandigarh, India; 4 Department of Psychology, Punjabi University, Patiala, India; 5 Indian Institute of Technology Ropar, Rupnagar, India; 6 Swiss Research Institute for Public Health and Addiction, Universitat Zurich, Zürich, Switzerland

**Keywords:** alcohol use, audit, brief advice, brief intervention, digital, screening

## Abstract

**Background:**

Drinking among college students in India is rising, influenced by social and cultural shifts. This study aimed to classify drinking behaviors among underage students using Latent Class Analysis (LCA) and assess the effectiveness of digital screening and brief intervention (DSBI) and brief advice (DSBA) across identified classes.

**Methods:**

This was a secondary analysis of a two-stage cluster randomized trial that screened 693 college students (age 18–22 yrs) with the Alcohol Use Disorders Identification Test (AUDIT). LCA was employed to identify latent classes in a main sample (N = 693, AUDIT ≥1) and a subsample of enrolled participants (N = 548, AUDIT scores 8-19). Model fit was assessed using AIC, BIC, and log-likelihood values, with a 3-class solution preferred for its interpretability and fit. A General Linear Model (GLM) analyzed the effectiveness of digital interventions over 3 and 6 months.

**Results:**

Three latent classes emerged from the main sample: “recreational drinkers” (minimal issues), “hidden problem drinkers (limited family concerns),” and “problem drinkers” (higher family concerns). In addition to the “hidden problem drinkers”, the subsample comprised “emerging” and “observable” *problem drinkers*. Significant reductions in AUDIT scores were observed across all classes from baseline to 3 months, which stabilized by 6 months. A significant interaction effect was found between time and latent class [F (4) =14.60, p<.0001], “observable problem drinkers” outperforming the two other classes.

**Conclusions:**

Underage drinking represents three latent classes. Digital interventions can effectively reduce alcohol use across all classes of underage college problem drinkers.

## 1 Introduction

Alcohol consumption among college students is a critical public health concern globally, with significant implications for health and well-being. The National Survey on Drug Use and Health (NSDUH) reports that nearly 50% of college students engage in drinking behaviors and nearly one in three engaged in heavy episodic drinking in the past month (1). Such patterns are linked to both acute harms (injuries, occasionally death) and elevated risk of longer-term outcomes, including alcohol use disorders, chronic illness, and academic difficulties (2). Systematic research on the frequency of alcohol use and heavy drinking among underage college students is limited in India. Nevertheless, regional studies show more than 50 percent of Indian college students have used alcohol in their lifetime (3, 4), and nearly one-third have problem drinking (4).

Cultural norms surrounding alcohol consumption in India are complex and evolving. Traditional perspectives often stigmatize alcohol use, yet changing social dynamics, fueled by urbanization and globalization, are altering these attitudes among the young people (5, 6). Among young people, studies from college students in Kerala (a southern Indian state) show that alcohol use and high-risk drinking are more common among men, non-Muslim students, urban residents and those embedded in peer networks where drinking is normalised (7). Recent population-based work among young men in Bihar and Uttar Pradesh (northern and eastern Indian states) further indicates that community-level acceptability, parental drinking, and broader socio-economic context are important determinants of alcohol use, over and above individual risk factors (8). Taken together, these findings suggest that even within a single country and age band, young drinkers are embedded in diverse cultural milieus that shape their motives, and may affect the patterns, and consequences of drinking. Research indicates that college students face unique risks associated with alcohol use, including peer pressure and accessibility, leading to increased consumption rates (9). A recent qualitative study from India applied social norms theory to understand alcohol and illicit drug use among college students. Drawing on in-depth interviews and focus group discussions, the study highlights how the “Normative Components” of social norms such as media representations and supportive environments influence individual drinking behavior. The authors illustrate the interplay of descriptive (e.g. perceived peer use, media portrayals and campus drinking environments) and injunctive norms (e.g. family disapproval, institutional rules and legal sanctions), social and health consequences awareness, and the collective impact on college students’ drinking decisions (10).

In general, culture, context, and identity influence alcohol use, misuse, and heavy drinking. The definitions of “moderation” and “drunkenness” may vary across cultures owing to the different group expectations, and culture may mitigate the “negative consequences” of alcohol use (11). Cultural norms around drinking may influence individual identity, which in a way can influence drinking behavior; for instance, Latino men who identify alcohol use with strong masculinity or “machismo” are more prone to heavy drinking and alcohol use disorder (12, 13). Stronger family values, especially among those who share negative views on alcohol with their family, and a stronger attachment to the family might reduce drinking behavior among young people (12). We also argue that a negative family and societal attitude toward alcohol and people who use alcohol may encourage the user to hide drinking and associated problems/behaviors to minimize stigma and discrimination (14). Similarly, in the Indian context, a patriarchal social order in which alcohol use is more tolerated among men than women; strong expectations around family honour (izzat) and respectability; and a collectivist orientation in which individual behaviour is judged in relation to its impact on the family’s reputation (15). For young men, drinking may be linked to displays of masculinity, peer bonding, and modernity, whereas for women, drinking often remains strongly disapproved of and may be hidden (10). At the same time, families may condemn ‘visible’ heavy drinking while tacitly accepting occasional or concealed use. These dynamics can encourage people, especially those from more conservative families or communities, to conceal their drinking and associated harms to avoid stigma and protect the family’s honour (10, 15).

Despite growing evidence of alcohol use among Indian young adults, systematic assessment of drinking patterns and related harms in this group remains limited. The Alcohol Use Disorders Identification Test (AUDIT), developed by the WHO, is a validated screening tool that assesses alcohol consumption, drinking behaviors, and alcohol-related problems. This instrument has been effectively utilized in diverse cultural settings, including India, to identify hazardous drinking patterns (3, 16). AUDIT can also identify cross-cultural differences in drinking behaviors (17). The cultural and contextual diversities in drinking have prompted researchers to categorize people who drink into relatively homogenous groups. In a general-population sample from the United States, Slater et al. identified five classes- nondrinkers, light drinkers, moderate drinkers, episodic heavy drinkers, and regular heavy drinkers, that differed systematically in psychosocial characteristics and health behaviours, illustrating how typologies can flag groups at particular risk (18). Similarly, Rouillier et al. reported distinct beverage-based patterns among French men (e.g. ‘low drinkers’, ‘high-quality wines’, ‘beer and cider’, ‘digestives’, ‘local wines’, ‘table wines’) (19). In a community sample of 16–20-year-olds in the United States, Reboussin et al. used latent class analysis to distinguish youth with low-risk alcohol use from those engaging in frequent heavy drinking accompanied by multiple alcohol-related problems (20). A study from England, among the adult general population showed a six latent class model explained the drinking behavior best, from mild consumption with no problems to heavy consumption with multiple injuries, memory blackouts and social pressure to cut down, with clear differences in mental-health outcomes between classes (21). The latter study used AUDIT for the latent class analysis (LCA). In sum, we observe variations in the classification of peoples’ drinking behavior across countries and age groups and LCA can combine information on frequency and quantity of drinking, dependence-like features, and adverse consequences to derive typologies that are both clinically interpretable and sensitive to contextual differences.

Therefore, it is justified to use LCA to explore the typology of alcohol use among underage Indian college students and to examine whether these empirically derived patterns show differential responses to a digital screening and brief intervention. Identifying distinct classes can inform how digital SBI is tailored (e.g. more intensive content or booster contact for higher-risk/problem classes, preventive and skills-building content for lower-risk classes) and how limited campus health resources are prioritized. Ultimately, the findings will contribute to the growing body of literature on alcohol use among young adults in India and inform public health initiatives aimed at promoting responsible drinking behaviors.

We used the data collected for a cluster randomized trial to determine the effectiveness of digital screening and brief intervention (DSBI) in college students with underage drinking (22). The hypothesis posited that the variability in alcohol-related behaviors in underage drinkers could be categorized into distinct patterns that reflect both alcohol consumption and associated problems. Specifically, we aimed (i) to identify latent subgroups of underage college drinkers in an Indian state based on item-level AUDIT responses, and (ii) among those eligible and enrolled in the randomized trial, to evaluate whether these latent subgroups differ in their response to a digital screening and brief intervention over 3 and 6 months.

## 2 Methods

### 2.1 Data source

The dataset used for the LCA was obtained from a state-wide cluster randomized trial designed to evaluate the effectiveness of a DSBI for alcohol misuse among college students in Punjab, India (22). We followed a two-stage cluster randomization- ten out of the 22 districts were selected in the first stage and 3–4 colleges were selected from each distinct in stage 2. Principles of probability proportionate to sample size were used in each stage. 1240 students were screened in the main trial and 548 participants were enrolled from 40 colleges, utilizing the AUDIT to assess alcohol consumption and related problems.

The primary trial was approved by the Institute Ethics Committee (I E C-0 8/2 0 1 9-1 3 1 2) and was registered at the Clinical Trial Registry India (Reference No. CTRI/2021/04/033287). A written informed consent was obtained from all participants and the trial conformed to the provisions of the Declaration of Helsinki.

### 2.2 Participants

Participants were undergraduate and postgraduate college students aged 18–22 years recruited as part of a state-wide cluster randomized trial of digital screening and brief intervention for alcohol and other substance use. For the present analysis, from the 1240 screened students, we first defined a main LCA sample comprising all students who reported any alcohol use in the past three months and had a non-zero baseline AUDIT score with complete item data. This group (N = 693; AUDIT ≥ 1) was used to describe the latent structure of underage drinking in the screened college population.

Within this main sample, a nested subsample of students met the eligibility criteria for the parent randomized trial (baseline AUDIT score between 8 and 19, indicating hazardous/harmful but non-dependent use, and provision of informed consent for trial enrolment) (22). There were no participants with an AUDIT score of more than 19. These participants (N = 548; AUDIT range 8–19) were randomized to DSBI versus digital screening and brief advice (DSBA) and followed up at 3 and 6 months. Importantly, all 548 participants are contained within the main sample of 693 drinkers; the trial subsample is therefore fully nested within, and not independent of, the main LCA sample.

### 2.3 Measures

#### 2.3.1 Demography and family history

Participants reported their age (in years) and gender (male, female, other) as part of the baseline survey. Participants were also asked whether any first-degree relatives (parents or siblings) had a history of alcohol use problems (yes/no), which was coded as a binary variable (“family history of alcohol use”).

#### 2.3.2 Alcohol use: AUDIT

Alcohol use and alcohol-related problems were assessed with the AUDIT, a 10-item screening instrument developed by the World Health Organization to detect hazardous and harmful alcohol use and possible dependence (16). Items 1–3 assess consumption (frequency of drinking, typical quantity per occasion, frequency of heavy episodic drinking), items 4–6 assess dependence-like features (impaired control, failure to meet expected roles, morning drinking), and items 7–10 assess adverse consequences and concerns (guilt or remorse, blackouts, alcohol-related injuries, and others expressing concern or suggesting the person cut down). Each item is scored from 0 to 4 according to standard WHO guidelines, yielding a total score from 0 to 40, with higher scores indicating more hazardous or harmful use and greater alcohol-related problems.

For the parent trial, baseline AUDIT scores of 8–19 were used to define hazardous/harmful but non-dependent drinking and to determine eligibility for randomization into the digital intervention arms. In the present analysis, we used the item-level AUDIT scores (0–4 per item) as ordinal indicators in the latent class models, and AUDIT total scores at baseline, 3 months, and 6 months as continuous outcome measures of alcohol use over time.

#### 2.3.3 Alcohol-related behaviours and problems

In addition to the AUDIT items, several single-item self-report questions captured specific alcohol-related behaviours and problems. These were used as correlates of latent class membership. All items were coded as binary variables (0 = no, 1 = yes) based on participants’ endorsement.

- Physical violence: whether they had “ever been involved in a physical fight or violence after drinking alcohol.”
- Intoxicated driving: whether they had “ever driven a motor vehicle after drinking enough to feel intoxicated.”
- Using alcohol to relax: whether they “used alcohol to relax or feel better when stressed or upset.”
- Offered alcohol by others: whether they “had been offered alcohol by friends, peers, or family in situations where they were not planning to drink.”
- Difficulty saying no to alcohol: whether they “found it difficult to say no when offered alcohol by others.”

#### 2.3.4 Physical and mental health variables

Participants were asked whether they had any chronic medical conditions (e.g. diabetes, hypertension, asthma or other long-term illnesses) currently under treatment (yes/no). We also assessed history of mental health problems using a single yes/no item (“Have you ever had any mental health problem such as depression, anxiety, or other psychological difficulties diagnosed by a health professional?”). In addition, participants indicated whether they had “ever consulted a psychiatrist or other mental health professional” for emotional or psychological concerns (yes/no). These variables were included as indicators of broader health vulnerability and were used as correlates of latent class membership.

### 2.4 Latent class analysis and further analysis

The LCA was conducted using the Gaussian Mixture Models with the AUDIT scores to identify distinct patterns of alcohol use. In a Gaussian mixture model, the observed distribution of a continuous variable is represented as a weighted combination of several normal (Gaussian) distributions, each corresponding to a latent subgroup or “component.” LCA extends this logic to categorical or ordinal indicators, estimating a small number of unobserved classes that differ in their item-response probabilities. In the present study, we used LCA with ordinal AUDIT items to identify qualitatively distinct subgroups of drinkers with different patterns of consumption and alcohol-related problems. LCA models with 2, 3, and 4 class solutions were specified and compared, with the optimal number of classes determined based on criteria such as the Akaike Information Criterion (AIC), Bayesian Information Criterion (BIC), and model interpretability. Given our sample size, we considered models with two to four classes *a priori*. Extracting a larger number of classes would have resulted in very small groups with unstable parameter estimates and limited practical utility. The models were estimated using maximum likelihood estimation, which facilitated the identification of class membership probabilities and item response probabilities associated with each class. Upon identifying the optimal model, the characteristics of each class were examined concerning AUDIT scores. The LCA was performed using Python along with the following libraries to generate the visualizations: Pandas for data manipulation and analysis, Seaborn for creating statistical data visualizations, and Matplotlib for plotting and customizing visualizations.

Furthermore, associations between latent classes and demographic factors, including age and gender, as well as current mental health status, were explored using chi-square tests and ANOVA.

For the intervention analyses (N = 548), we first assigned each participant to their most likely latent class based on posterior probabilities from the LCA fitted in this subsample. We then examined changes in AUDIT total scores over time using general linear model (GLM), with fixed effects for time (baseline, 3 months, 6 months), intervention group [digital screening and brief intervention (DSBI) vs digital screening and brief advice (DSBA)], and latent class, as well as their interaction terms (particularly the group × time and group × time × class interactions). Random intercepts for participants accounted for the repeated-measures structure of the data. This allowed us to test whether the effect of the digital intervention on AUDIT scores over time differed across latent subgroups of drinkers.

We used the Statistical Package for Social Sciences (SPSS, v 21) for this analysis and visualization of chi-square tests, ANOVA, and GLM.

## 3 Results

### 3.1 Sample

In the main sample (N = 693), the mean age of the participants was 19.22 (Standard Deviation 1.28) years. There were 446 (64.4%) boys and 247 (35.6%) girls. Participants were enrolled from ten districts and forty colleges. The trial subsample had a mean age of 19.18 (SD 1.26) years and the sex distribution was also similar, 342 boys (62.4%) and 206 girls (37.6%). The minimum AUDIT score for the full LCA sample (N = 693) and trial subsample (N = 548) was 1 and 8, and the maximum AUDIT score for the full samples was 15.

### 3.2 Latent class analysis: model fit statistics

For both datasets (N = 693 and N = 548), latent class models were evaluated across 2-, 3-, and 4-class solutions to determine the best fit based on AIC, BIC, entropy, and log-likelihood values. See **Table 1**.

**Table 1:** Model Fit Statistics for the Latent Class Analysis.

Number of classes	AIC	BIC	Entropy	Log-likelihood
N= 693 main sample				
2	-192.9565803	401.9183496	-1.00E-10	227.4782902
3	-6029.868838	-5135.285928	-1.00E-10	3211.934419
4	-5470.172926	-4275.882036	0.000590719	2998.086463
N= 548 sub-sample				
2	1936.138945	2500.261007	-1.00E-10	-837.0694724
3	-1304.951215	-456.6149837	-1.00E-10	849.4756076
4	-2593.611726	-1461.061325	0.000190947	1559.805863

Main sample: Class 1= recreational drinkers, Class 2= problem drinkers, Class 3= hidden problem drinkers.

Sub-sample: Class 1 = observable problem drinkers, Class 2 = hidden problem drinkers, Class 3= emergent problem drinkers.

We first describe the latent class structure of underage drinking in the main LCA sample (N = 643), which includes all screened students who reported recent alcohol use and had a non-zero AUDIT score. We then turn to the trial subsample (N = 548), which is nested within this main sample and comprises students with hazardous/harmful but non-dependent drinking who were enrolled in the randomized digital intervention trial. In this subsample, we examine whether similar latent classes are observed, association of the latent classes with measured demographic variables, variables indicating alcohol-related behaviors/problems, and mental and physical health issues. In the trial sub-sample, we also examine whether intervention effects on AUDIT scores over time differ across classes.

#### 3.2.1 LCA of the main sample (N = 693)

The analysis of the sample used alcohol in the last three months (N = 693) generates a 3-class solution as the optimal choice, achieving a low AIC (-6029.87) and BIC (-5135.29), and a high log-likelihood of 3211.93. The entropy (-1.00E-10) in this solution remains low, reflecting sufficient class distinctiveness without the need for additional complexity. Although the 4-class solution produced similar fit improvements, the 3-class model offers a more streamlined interpretation, preserving analytical clarity and providing insights that align with the broader population trends observed. As such, the 3-class model is the preferred solution for the main sample, offering an effective balance of statistical fit and practical interpretability.

**Figure 1:**
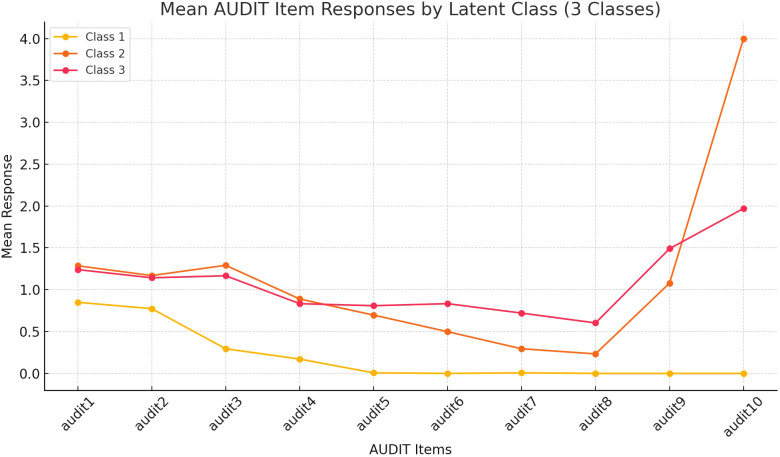
Mean AUDIT Item Responses for 3-Class Model in the Main Sample (n=693). This figure shows the mean response patterns for AUDIT items across three latent classes identified in the analysis of all participants (n=693) who used alcohol in the last 3 months. Each class represents a distinct subgroup of participants, with Class 1, Class 2, and Class 3 displaying unique response trends across the 10 AUDIT items. Class 1= recreational drinkers, Class 2= problem drinkers, Class 3= hidden problem drinkers.

Class 1 consistently exhibits the lowest mean responses across most items, representing minimal or no alcohol-related problems. Class 3 stands out with higher scores on the items depicting severe health effects (items 6 to 8), but intermediate scores on items 9 and 10. While Class 2 has consumption scores aligned with Class 3 and the scores on the items on health effects are intermediate, the score on item 10 designed to detect concerns from others is the highest. However, the score on item 10, designed to capture external concerns, Class 2 has a very high score. We label Classes 1, 2, and 3 as “recreational drinkers,” “problem drinkers”, and “hidden problem drinkers.” See **Figure 1**.

#### 3.2.2 LCA of the trial sub-sample (N = 548)

For the subsample (N = 548) of people enrolled in the trial with AUDIT-scores indicating at least harmful or hazardous alcohol use (AUDIT 8-19), a 3-class latent class analysis model was chosen based on its optimal balance of fit statistics and interpretability. The 3-class solution provides a marked improvement over the 2-class model, with a significant reduction in AIC (-1304.95) and BIC (-456.61), alongside a high log-likelihood of 849.48. The entropy score, while minimal (-1.00E-10), suggests that class separation is adequately captured for the dataset’s characteristics. While the 4-class model showed slightly lower AIC and BIC, the additional complexity did not significantly enhance interpretability or class separation. Thus, the 3-class model provides a simpler, robust solution that effectively captures the main latent structures within this subsample.

**Figure 2:**
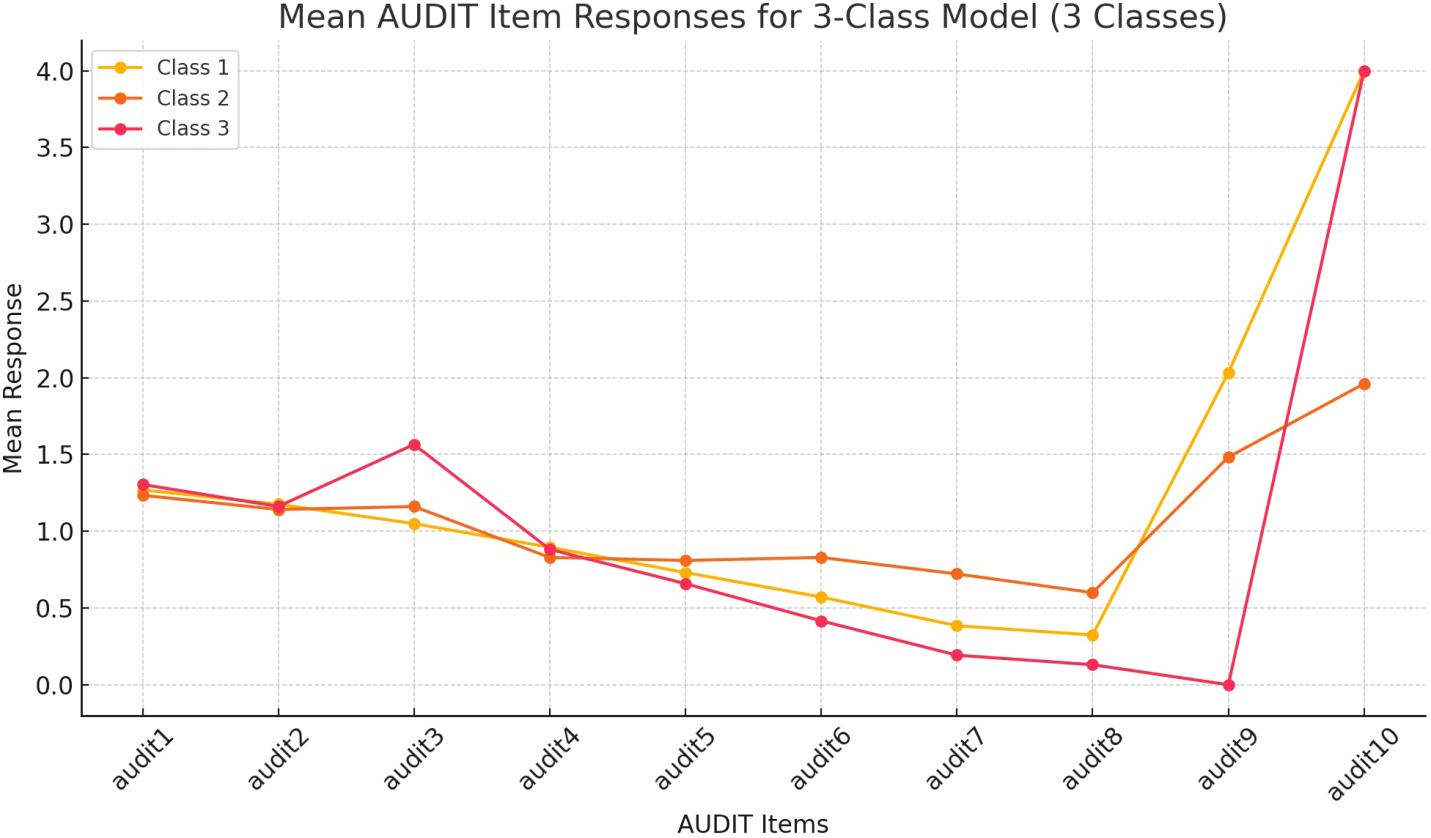
Mean AUDIT Item Responses for 3-Class Model in the Nested Sub-sample (n=543). This figure illustrates the response trends across AUDIT items for a three-class latent model in the analysis of participants (n=543) who scored between AUDIT 8-19, i.e., had harmful or hazardous alcohol use. The lines highlight differences in the mean response patterns between the three latent classes, indicating varying levels of alcohol-related behaviors and experiences. Class 1 = observable problem drinkers, Class 2 = hidden problem drinkers, Class 3= emergent problem drinkers.

The subsample of 548 participants (AUDIT 8-19) reveals distinct response patterns across the latent classes, with notable differences in AUDIT items 8, 9, and 10, and minimal separations between Class 1 and 2 in the consumption items (AUDIT 1-3). Class 1 generally displays the lowest mean scores across consumption items, intermediate scores in the items on health effects, and highest scores on items 9 (direct harms to self and others) and 10 (concerns expressed by others). Class 2 does not separate it from Class 1, particularly in frequency and quantity of drinking. However, Class 2 has higher scores on health effects and intermediate scores on items 9 and 10. Class 3 has the highest mean scores in consumption items (especially, in heavy drinking). Although the scores of Classes 3 on the items designed to detect health effects and direct harm to self and others are the lowest among the three, the score in item 10 is the highest. Class 2 in this sub-sample corresponds with Class 3 of the main sample, the “hidden problem drinker.” There is no corresponding “recreational drinker” class in the sub-sample. Class 1 and 3 represent the “problem drinkers” class (Class 2) of the main sample. We label these classes as “observable problem drinkers” and “emerging problem drinkers,” respectively. See **Figure 2**.

### 3.3 Comparison of latent classes (sub-sample, N = 548)

In the subsample of 548 participants, a comparative analysis of latent classes was conducted across various demographic and behavioral variables. Age differed significantly among the latent classes, F (2,545) =5.24, p=.006 with Class 2, i.e., “hidden problem drinkers,” (M = 19.46 yrs, SD = 1.37) having a slightly higher mean age than Class 1, i.e., “observable problem drinkers,” (M = 19.07, SD = 1.23) and Class 3, i.e., “emerging problem drinkers,” (M = 19.07, SD = 1.24). The AUDIT total scores also showed a significant difference, F (2,545) = 84.99, p <.001, with Class 1 (“observable problem drinkers“) reporting the highest mean score (M = 12.00, SD = 1.70) compared to Class 2, i.e., “hidden problem drinkers,” (M = 11.44, SD = 1.35) and Class 3, i.e., “emerging problem drinkers,” (M = 10.09, SD = 1.34), indicating varying levels of alcohol use severity across classes.

Family history of alcohol use was associated with latent class membership, χ2(2, N = 548) =8.78, p=.012, with Class 2 (“hidden problem drinkers”) showing a higher percentage of positive family history (37.7%) compared to Class 1 (“observable problem drinkers”) (26.4%) and Class 3 (“emerging problem drinkers”) (23.8%). For getting into trouble due to alcohol use, there was also a significant difference among classes, χ2(2, N = 548) =6.51, p=.039, with Class 2 (12.5%) more frequently reporting trouble related to alcohol use than Class 1 (5.7%) and Class 3 (6.3%).

No significant differences were found among the latent classes regarding chronic medical disease, psychological disorders, psychiatrist visits, using alcohol to relax, being offered alcohol, difficulty saying no, or intoxicated driving. See **Table 2**.

**Table 2:** Comparison of demographic and behavioral variables between the latent classes (N = 548).

Variables	L1 (N = 208)	L2 (N = 151)	L3 (N = 189)	F/χ2 value	P- value
Age in years (Mean ± SD)	19.07 ± 1.226	19.46 ± 1.365	19.07 ± 1.240	5.237	0.006
AUDIT Total (Mean ± SD)	12.00 ± 1.698	11.44 ± 1.349	10.09 ± 1.336	84.990	0.000
Family history of alcohol use	55 (26.4%)	57 (37.7%)	45 (23.8%)	χ2 = 8.777	0.012
Chronic medical disease	11 (5.2%)	8 (5.2%)	9 (4.7%)	χ2 = 0.072	0.965
Psychological disorder	4 (1.9%)	3 (1.9%)	3 (1.5%)	χ2 = 0.093	0.955
Psychiatrist visit	11 (5.2%)	13 (8.6%)	7 (3.7%)	χ2 = 3.870	0.144
Alcohol to relax	21 (10%)	19 (12.5%)	19 (10.05%)	χ2 = 0.716	0.699
Offered alcohol	54 (25.9%)	52 (34.4%)	59 (31.2%)	χ2 = 3.155	0.207
Difficult to say No	66 (31.7%)	46 (30.4%)	64 (33.8%)	χ2 = 0.468	0.791
Getting into trouble because of alcohol use	12 (5.7%)	19 (12.5%)	12 (6.3%)	χ2 = 6.512	0.039
Intoxicated driving	10 (4.8%)	16 (10.5%)	15 (7.9%)	χ2 = 4.321	0.115

Class 1 = observable problem drinkers, Class 2 = hidden problem drinkers, Class 3= emergent problem drinkers.

### 3.4 Response to digital alcohol brief intervention

In examining the effects of digital interventions (DSBI and DSBA) across three latent classes over two follow-up points (3 and 6 months), several significant results were found.

A main effect of time was observed, F (2) =4224.04, p<.0001, with a very large effect size (partial η² = .940), indicating substantial changes across time points in the outcome measure across all groups and classes. Additionally, a significant interaction effect was found between time and latent class, F (4) =14.60, p<.0001, with a partial η² of.051, suggesting that the latent classes responded differently over time. Class 1, the “observable problem drinkers,” although had the highest initial AUDIT score, reached the lowest AUDIT severity at 6 months. Although there was a significant AUDIT reduction among the “observable problem drinkers” between follow-up at 3 and 6 months, no such reductions were seen in the other two categories (“hidden problem drinkers” and “emerging problem drinkers”).

No significant interaction effects were observed for the time by group, F (2) =2.41, p=.91, indicating that the intervention groups did not differ in their response patterns over time. Furthermore, the three-way interaction of time, latent class, and group were also non-significant, F (4) =0.006, p=.531. This suggests that the combination of latent class and intervention type did not significantly influence the changes observed over time. See **Table 3**; **Supplementary Figures 1, 2**.

**Table 3:** Interaction models (N = 543).

Effect	F- value	Degree of freedom (Df)	P-value	Effect size/partial η^2^
Time	4224.035	2	0.0001	0.940
Time x latent class 3	14.603	4	0.0001	0.051
Time x Group	2.409	2	0.91	0.009
Time x latent class 3 x group	0.006	4	0.531	0.003

## 4 Discussions

This study examined alcohol use patterns among underage college students in India, focusing on identifying latent classes of alcohol consumption behaviors and assessing the impact of digital screening and brief intervention (DSBI) on these distinct groups. Our results revealed three distinct latent classes in both the main sample (N = 693) and the subsample (N = 548), labeled as “recreational drinkers,” “problem drinkers,” and “hidden problem drinkers” in the main sample, and as “observable problem drinkers,” “emerging problem drinkers,” and “hidden problem drinkers” in the subsample. The “hidden problem drinker,” common to both LCA, reflects a class reporting substantial alcohol-related problems on the AUDIT but relatively fewer indicators of visible trouble or external confrontation, suggesting that their difficulties may be less apparent to family members or institutional gatekeepers. These findings highlight the heterogeneity of drinking behaviors among college students, underscoring the need for tailored interventions.

The LCA conducted on the main sample (N = 693) revealed that “recreational drinkers” (Class 1) exhibited the lowest mean responses on most AUDIT items, indicating minimal alcohol-related issues. The “hidden problem drinkers” (Class 3) had consumption levels similar to the “problem drinkers” (Class 2) but “problem drinkers” scored particularly high on item 10, reflecting external concerns related to their drinking behaviors. This pattern suggests that the “hidden problem drinkers” may experience less social and familial pressure to moderate their drinking or can conceal it, possibly due to stigma or the desire to avoid judgment (15). The “problem drinkers” showed higher scores than “recreational drinkers” on items associated with both consumption and health effects, indicating more severe drinking behaviors and related health consequences. This conjecture was supported by the highest score on item 10, indicating the potential impact of alcohol use on relationships and health. A higher score on this item suggests that others have recognized problematic drinking, which can be an indicator of more severe alcohol-related issues. However, the ‘problem drinkers’ experienced less severe health and direct harmful effects of alcohol than the ‘hidden problem drinkers.’ One plausible explanation, consistent with social norms and social control theories, is that these individuals may be embedded in more receptive social circles, i.e., family members, peers, or partners, who notice visible drinking-related problems and raise concerns earlier, thereby prompting some degree of behaviour change or help-seeking (10, 12).

A study from Great Britain used AUDIT to identify latent classes, revealing six distinct groups ranging from mild to heavy consumption with varied negative consequences, including classes marked by injury, social pressure to cut down, and memory loss (21). Similarly, our study identified three distinct classes, i.e., “recreational drinkers,” “hidden problem drinkers,” and “problem drinkers,” reflecting a range of alcohol use severity. However, while the Great Britain study found six distinct types, our three-class solution reflects the relatively simpler drinking patterns seen in younger, underage Indian college drinkers. Although we did not directly assess cultural or attitudinal factors, the distinction between ‘observable’ and ‘hidden’ problem drinkers is compatible with the hypothesis that cultural norms, stigma, and family expectations shape how drinking-related problems become visible to others. This remains a contextual interpretation and requires confirmation in studies that explicitly measure these constructs. This was supported by previous studies from the US and Norway (20, 23).

In the subsample, while similar patterns emerged, some differences were observed. The lack of a clear “recreational drinker” class in this subsample may reflect the fact that participants in this group had higher AUDIT scores overall due to the trial’s enrollment criteria (22). Instead, we observed a class labeled as “observable problem drinkers,” characterized by high initial scores, especially on items indicating direct harms and external concerns, but demonstrating a significant decrease in AUDIT scores over time. This suggests that digital interventions may be particularly effective for students whose drinking behaviors and related consequences are visible, possibly because these individuals are more motivated to change or more receptive to accessible interventions. It might also suggest those with visible problems are more amenable to the “feedback (24, 25).” The responsiveness of the “observable problem drinkers” to intervention indicates a critical area for targeted, early intervention, as these individuals are more likely to benefit from brief, behavior-focused efforts. Nevertheless, the response to digital interventions in all three classes and across the brief intervention and brief advice frameworks supported the effectiveness of SBI among underage college drinkers (22).

The “hidden problem drinkers” have a higher family history of alcohol use and a history of getting into trouble due to alcohol use (indicative of socially inappropriate use). The use of alcohol by other family members may explain the higher “acceptance” of alcohol in the family, which in turn may explain limited concerns expressed by them despite higher physical and social harms among the “hidden problem drinkers” class (26). The higher age of the “hidden problem drinkers” may indicate developmental factors influencing drinking behaviors or the onset of drinking-related problems (27).

The response to interventions (DSBI vs. DSBA) did not significantly differ between the latent classes, at least three months post-intervention. We believe the following might be responsible for the strong and cross-cutting effect of both types of digital interventions- a) “abstinence-focused” cultural norm (12) and b) the introduction of a novel digital intervention (for the exposed population) to facilitate the reduction in drinking in an environment where institutional screening and help for those with problematic drinking is lacking. The abstinence-focused norms might have created a secular drift towards a reduction in drinking across classes of alcohol users (28). Previous research has demonstrated how intervention characteristics like novelty and subject characteristics such as those who are unfamiliar with such interventions show a greater effect on behavior change (29).

### 4.1 Public health, policy and practice implications

The observed reductions in AUDIT scores across all latent classes underscore the potential effectiveness of early, intensive intervention efforts in curbing alcohol use among all types of underage college students. The pattern suggests that an initial digital intervention phase can yield substantial short-term improvements. However, the stabilization in scores after 3 months indicates that these gains may not be sustained without ongoing support, highlighting the need for a phased intervention model in policy and practice. Implementing periodic follow-ups, booster sessions, or continued access to digital resources could help maintain these early improvements over the long term (30).

Recognizing the diversity in drinking behaviors among underage college drinkers in India highlights the need for targeted prevention and intervention approaches. For example, “observable problem drinkers” showed a strong response to digital interventions, suggesting that accessible and scalable digital brief interventions could be implemented within college settings for this group. Universities could integrate such interventions into their health services or make them available as part of a student wellness initiative, reaching students who are visibly at risk (30).

“Hidden problem drinkers” presents a unique challenge. Without targeted support, hidden problem drinkers risk developing more entrenched patterns that can lead to serious long-term consequences, such as academic issues, mental health challenges, and social isolation (31). For this group, digital interventions could play a crucial role in providing discrete, accessible help without the stigma often associated with in-person services (32, 33). Our findings also indicated the potential of engaging the family of “hidden problem drinkers.”

## 5 Limitations

The sample was restricted to college students in a single state in India, limiting the generalizability of findings. Given the recruitment of participants with elevated AUDIT scores (8–19) for the intervention, regression towards mean might look like a plausible explanation for improvement. However, several findings in our study indicate that the observed changes might not be attributable to RTM. First, participants were recruited from diverse college settings across multiple districts, ensuring heterogeneity in drinking behaviors and demographic characteristics. This diversity reduces the likelihood that our results reflect a uniform statistical artifact. Second, the significant differences in the trajectories of latent classes, such as the notable improvement observed among “observable problem drinkers,” suggest a targeted effect of the intervention rather than a generalized regression. RTM would predict similar declines across all classes, but our results demonstrated class-specific variations, particularly in items related to health and social consequences. Furthermore, the active engagement of participants with tailored digital interventions likely introduced external influences beyond natural statistical variation. We also observed stabilization of AUDIT scores after three months, a pattern consistent with intervention effects. While the lack of a general population sample limits broader generalizability, this study focused on a high-risk group where tailored interventions are most needed.

Other limitations were, data on alcohol consumption were self-reported, introducing potential reporting biases, especially given the stigma surrounding drinking in India. The follow-up period was limited to 3 and 6 months, which may not capture the long-term sustainability of intervention effects. When examining associations between covariates and latent class membership, we used participants’ most likely class in regression models rather than a three-step approach that accounts for classification uncertainty. This “hard-classification” method can bias class–covariate associations and underestimate standard errors (34, 35). However, in our data, high posterior probabilities and good entropy indicated clear class separation, so any bias is likely to be small. Finally, this was a secondary analysis; hence, it should be considered exploratory. Previous studies indicated an association of latent classes with socioeconomic status and education, and negative mental and physical health consequences (21, 23, 36, 37). We had only a few demographic and drinking variables for comparing between the latent classes. Moreover, some variables (e.g., mental health consequences) were measured with a single self-reported question. Hence, negative findings for these associations in our study should not be interpreted as the absence of such associations. Future research with broader geographical samples, more objective alcohol consumption measures, and extended follow-up periods is recommended to strengthen these findings.

## 6 Conclusion

This study identified three distinct alcohol use patterns among Indian underage college students and demonstrated that digital interventions can effectively reduce AUDIT scores in all three classes, especially in the short term. Sustained and tailored interventions, particularly for “hidden problem drinkers,” are crucial to maintaining these reductions and preventing long-term alcohol-related harms.

## Data Availability

The raw data supporting the conclusions of this article will be made available by the authors, without undue reservation.
