# Supplementary material for: Patterns of alcohol use and response to digital brief interventions in college students: a secondary analysis of a cluster randomized trial
Source: Front Psychiatry. 2026 Jan 12;16:1732518. doi: 10.3389/fpsyt.2025.1732518 (PMC12833505; doi:10.3389/fpsyt.2025.1732518)
Supplement: Supplementary file 1 [file Table1.docx]

**Supplementary Figure 1**

**Change of AUDIT scores in three Latent Classes Across the three Time points in the DSBA group**

**
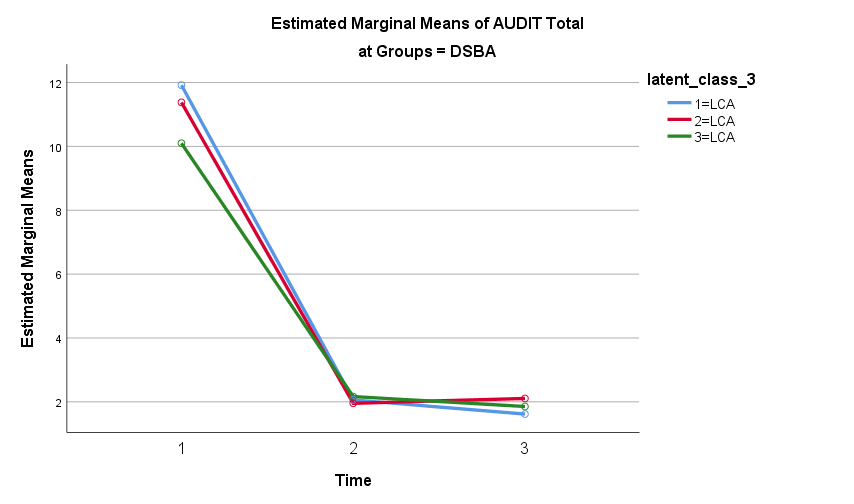
**

**Supplementary Figure 2**

**Change of AUDIT scores in three Latent Classes Across the three Time points in the DSBI group**

**
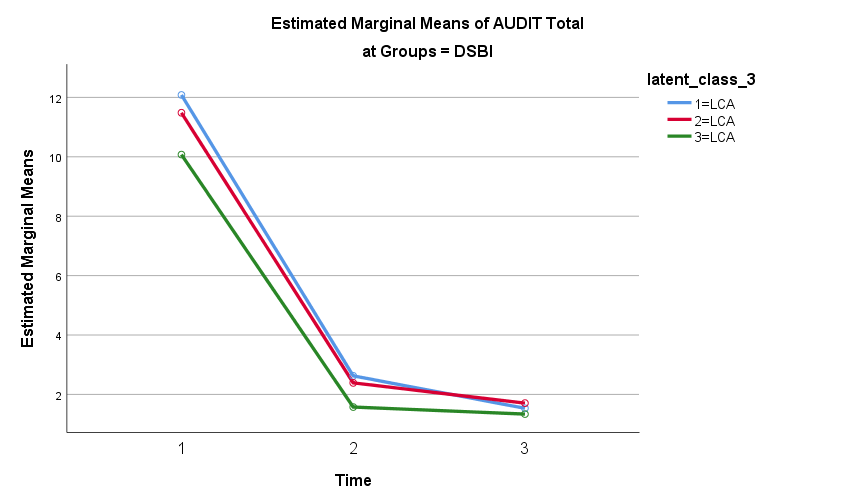
**
