# Supplementary material for: Patterns of alcohol use and response to digital brief interventions in college students: a secondary analysis of a cluster randomized trial
Source: Front Psychiatry. 2026 Jan 12;16:1732518. doi: 10.3389/fpsyt.2025.1732518 (PMC12833505; doi:10.3389/fpsyt.2025.1732518)
Supplement: Supplementary file 2 [file Table2.docx]

Alcohol use disorders identification test (AUDIT)

| **Questions** | **Scoring system** | | | | | **Your score** |
| --- | --- | --- | --- | --- | --- | --- |
|  | **0** | **1** | **2** | **3** | **4** |  |
| How often do you have a drink containing alcohol? | Never | Monthly or less | 2 to 4 times per  month | 2 to 3 times per  week | 4 times or more per week |  |
| How many units of alcohol do you drink on a typical day when you are drinking? | 0 to 2 | 3 to 4 | 5 to 6 | 7 to 9 | 10 or more |  |
| How often have you had 6 or more units if female, or 8 or more if male, on a single occasion in the last year? | Never | Less than monthly | Monthl y | Weekly | Daily or almost daily |  |
| How often during the last year have you found that you were not able to stop drinking once you had started? | Never | Less than monthly | Monthl y | Weekly | Daily or almost daily |  |
| How often during the last year have you failed to do what was normally expected from you because of your drinking? | Never | Less than monthly | Monthl y | Weekly | Daily or almost daily |  |
| How often during the last year have you needed an alcoholic drink in the morning to get yourself going after a heavy drinking session? | Never | Less than monthly | Monthl y | Weekly | Daily or almost daily |  |
| How often during the last year have you had a feeling of guilt or remorse after drinking? | Never | Less than monthly | Monthl y | Weekly | Daily or almost daily |  |
| How often during the last year have you been unable to remember what happened the night before because you had been drinking? | Never | Less than monthly | Monthl y | Weekly | Daily or almost daily |  |
| Have you or somebody else been injured as a result of your drinking? | No |  | Yes, but not in the last  year |  | Yes, during the last year |  |
| Has a relative or friend, doctor or other health worker been concerned about your drinking or suggested that you cut down? | No |  | Yes, but not in the  last year |  | Yes, during the last year |  |

**Total AUDIT score**
